# Comparative transcriptomics reveals the selection patterns of domesticated ramie

**DOI:** 10.1002/ece3.5271

**Published:** 2019-05-22

**Authors:** Kun‐Yong Huang, Ai‐Guo Zhu, Xiao‐Rong Chen, Ya‐Liang Shi, Qing Tang, Xiao‐Fei Wang, Zhi‐Min Sun, Ming‐Bao Luan, Jian‐Hua Chen

**Affiliations:** ^1^ Institute of Bast Fiber Crops, Chinese Academy of Agricultural Sciences/Key Laboratory of Stem‐Fiber Biomass and Engineering Microbiology Ministry of Agriculture Changsha China; ^2^ Institute of Bast Fiber Crops in Jiangxi Yichun China

**Keywords:** differentially expressed genes, domestication, Ka/Ks, phylogenetic relationship, ramie, selective pattern

## Abstract

Although domestication has dramatically altered the phenotype, physiology, and life history of ramie (*Boehmeria nivea*) plants, few studies have investigated the effects of domestication on the structure and expression pattern of genes in this fiber crop. To investigate the selective pattern and genetic relationships among a cultivated variety of ramie (BNZ: *B. nivea*, ZZ1) and four wild species, BNT (*B*. *nivea* var. *tenacissima*), BNN (*B. nivea* var. *nipononivea*), BNW (*B. nivea* var. *nivea*), and BAN (*B. nivea* var. *viridula*), in the section Tilocnide, we performed an RNA sequencing analysis of these ramie species. The de novo assembly of the “all‐ramie” transcriptome yielded 119,114 unigenes with an average length of 633 bp, and a total of 7,084 orthologous gene pairs were identified. The phylogenetic tree showed that the cultivar BNZ clustered with BAN in one group, BNW was closely related to BNT, and BNN formed a separate group. Introgression analysis indicated that gene flow occurred from BNZ to BNN and BAN, and between BAN and BNN. Among these orthologs, 2,425 and 269 genes underwent significant purifying and positive selection, respectively. For these positively selected genes, oxidation–reduction process (GO:0055114) and stress response pathways (GO:0006950) were enriched, indicating that modulation of the cellular redox status was important during both ramie fiber evolution and improvement. Two genes related to the suppression of flowering and one gene annotated as a flowering‐promoting factor were subjected to positive selection, probably caused by human manipulation. Additionally, five genes were homologs of those involved in abiotic stress tolerance and disease resistance, with higher expression levels in the cultivar BNZ than in the wild species. Collectively, the results of this study indicated that domestication has resulted in the upregulation of many genes involved in the abiotic and biotic stress responses, fiber yield, and plant growth of ramie.

## 1 INTRODUCTION

Ramie (*Boehmeria nivea* L. Gaud) is a traditional fiber crop that has been cultivated for thousands of years in China (Liu, Tang, Zhu, Tang, & Zheng, 2014). The section Tilocnide includes four subspecies, namely *B. nivea* var. *nivea* (BNW), *B*. *nivea* var. *nipononivea* (BNN), *B*. *nivea* var. *tenacissima* (BNT), and *B*. *nivea* var. *viridula* (BAN), among which domesticated varieties of *B*. *nivea* var. *nivea* have been bred (Wang, 1981; Chen, Pan et al., 2014). As an outcrossed perennial crop, the genetic background of ramie is complex because of interspecific hybridization among the four species of section Tilocnide. On the basis of the specific trends in stipule, leaf hairs, and the external shape of pollen, Zhang, Zheng, Zuang, and Zhao (1998) found that in terms of evolutionary kinship, BAN was most closely related to BNT, followed by BNW and BNN. Recently, based on cluster analysis of ramie morphological characteristics, Chen, Pan et al. (2014) showed that BNW and BNN were initially clustered together, then with BNT, and subsequently BAN. Recently, researchers have analyzed the genetic relationships among ramie varieties using molecular markers, such as RAPD (Meng, Liu, & Peng, 2009), SSR (Zhu et al., 2017), and ISSR (Liu, Wang, Wang, & Peng, 2011). Using SRAP markers, Liao et al. (2010) found that BNW was closely related to BNN, whereas the BNT formed another cluster with BAN. However, to date, there has been limited analysis of the phylogenetic relationships of section Tilocnide members based on molecular characteristics.

Domestication has dramatically altered the phenotypes, physiology, and life history of cultivated plants (Sauvage et al., 2017) and thereby offers an opportunity to analyze evolutionary forces acting at the genomic and transcriptome levels (Morrell, Buckler, & Ross‐Ibarra, 2012). Major domestication traits are a result of the modification of gene expression patterns, as identified in cotton (Rapp et al., 2010), maize (Hufford et al., 2012), sunflowers (Blackman et al., 2011), rice (Huang et al., 2012), and tomatoes (Koenig, Jiménez‐Gómez, & Kimura, 2013). By comparing the divergence in gene expression between maize and teosinte, Swanson‐Wagner, Briskine, and Schaefer (2012) detected more than 600 significantly differentially expressed genes and more than 1,100 genes with significantly altered co‐expression profiles. Lemmon, Bukowski, Sun, and Doebley (2014) demonstrated the evolution of *cis* regulation during maize domestication and observed a directional bias, whereby alleles of maize genes with *cis* differences showed higher expression more often than those of teosinte genes. With regard to ramie transcriptome studies, there have been numerous studies on transcriptome changes in ramie in response to biotic/abiotic stresses, at different developmental stages, and in different organs (Liu, Zhu, Tang, Yu, & Tang, 2013b; Zhu, Tang, Tang, & Liu, 2014; Liu, Zhu, Tang, & Tang, 2015; Yu et al., 2015; An et al., 2015; Zeng et al., 2016). However, little is known about the domestication of ramie. Previous studies (Wu et al. 2003; Liu et al., 2014) have reported that domesticated ramie has longer and thicker stems, thicker bark and fiber cells, and produces more fiber than the wild species. To characterize the selective patterns occurring during ramie domestication, Liu et al. (2014) calculated the nonsynonymous (Ka) and synonymous (Ks) nucleotide substitutions of orthologous genes of the transcriptomes of one ramie variety (ZZ1) and the wild accession QYZM and found that most of the selected genes were annotated in abiotic stress tolerance or disease resistance, whereas two genes were related to fiber yield. However, there is currently limited information available regarding gene co‐expression shifts between wild and cultivated ramie during the process of domestication.

In this study, we sequenced three different tissues from five individuals, during the flowering period: four wild relatives of ramie [BNN (*B*. *nivea* var. *nipononivea*), BAN (*B*. *nivea* var. *viridula*), BNT (*B*. *nivea* var. *tenacissima*), and BNW (*B. nivea* var. *nivea*)], and the ramie cultivar ZZ1 (BNZ). We constructed transcriptomes for all the varieties. We then identified orthologous genes based on BLAST results for amino acid sequences. Using these orthologous gene pairs, we determined Ka/Ks ratios and constructed a phylogenetic tree for the section Tilocnide. Furthermore, we analyzed the gene flow of the four wild relatives of ramie and the ramie cultivar ZZ1 using the software *TreeMix* (version 1.12; Pickrell and Pritchard 2012). In addition, we separately assembled the five transcriptomes and determined differences in gene expression between the cultivar BNZ and the four wild ramie species that have occurred during ramie domestication.

## 2 MATERIALS AND METHODS

### 2.1 Sampling, library construction and transcriptome sequencing

Four wild ramie species (BNW, BNN, BNT, and BAN) of section Tilocnide and the ramie cultivar *B*. *nivea*‐ZZ1 (BNZ) were used in this study. All species were grown in the experimental field of the Institute of Bast Fiber Crops, Chinese Academy of Agricultural Sciences, Changsha, China. The stems, leaves, and inflorescences were separately sampled during the flowering period and immediately frozen in liquid nitrogen and stored at −80°C until use. The total RNA of each sample was extracted using an E.Z.N.A. Plant RNA Kit (OMEGA Bio‐Tek) according to the manufacturer's protocol.

After RNA quantification and qualification, a total amount of 1.5 µg RNA per sample was used for RNA sequencing. Sequencing libraries were generated using a NEBNext® Ultra™ RNA Library Prep Kit for Illumina^®^ (NEB) following the manufacturer's recommendations, and index codes were added to attribute sequences to each sample. Sequencing was performed using the Illumina^TM^ 2,500 platform at Novogene Bio‐informatics Technology Co., Ltd., Beijing, China (www.novogene.cn). The raw sequencing reads have been deposited in the NCBI Sequence Read Archive (SRA, http://www.ncbi.nlm.nih.gov/Traces/sra) with accession numbers SRP150685: SRX4224255‐SRX4224269.

### 2.2 Transcriptome sequencing assembly

#### 2.2.1 Reconstruction of all and individual ramie transcriptomes

Transcriptome assembly was accomplished using Trinity (Grabherr, Haas, & Yassour, 2011) with default parameters. After removing redundant contigs from the library, the remaining contigs were assembled into unigenes, which represent the ramie transcriptome during the flowering period. Using the same method, the transcriptomes of each of the five individual ramie species were constructed. The unigenes in a library represent the individual ramie transcriptomes during the flowering period. In addition, a unigene bank for each sample was generated using the same method.

#### 2.2.2 Functional annotation

We carefully annotated unigenes using various bioinformatics approaches. The unigenes were initially searched against the Nr (NCBI nonredundant protein sequences), Nt (NCBI nonredundant nucleotide sequences), and Swiss‐Prot protein databases using local BLASTx with *E*‐value < 1E‐5. The Blast2GO program (Conesa et al., 2005) was used to derive GO annotations according to molecular function, biological process, and cellular component ontologies. In addition, unigene sequences were aligned to the COG (Clusters of Orthologous Groups of proteins) database to predict and classify possible functions. Furthermore, pathway assignments were carried out according to the Kyoto Encyclopedia of Genes and Genomes (KEGG) pathway database (Kanehisa & Goto, 2000) using BLASTx with *E*‐value < 1E‐10.

#### 2.2.3 Identification of orthologous genes groups, construction of a phylogenetic tree, and calculation of KA/KS

The orthologous genes of the five ramie transcriptomes were identified according to the method reported by Li et al. (2003). A total of 7,084 orthologous gene pairs were identified from the BLASTP results using Ortho MCL v2.0.3 (Li et al. 2003) with default parameters (a *p*‐value cutoff of 1e‐5).

We performed the coding sequence (CDS) alignment of one‐to‐one orthologous genes using Muscle (Edgar, 2004). On the basis of the BLAST results of orthologous genes, we constructed a phylogenetic tree based on the maximum‐likelihood method of the PhyML program, v2.4.4 (Guindon et al. 2003).

Homologous genes with a high Ka/Ks ratio are generally evolving under positive selection, which indicates that these genes are under heavy selection pressure, whereas values ≤ 1 are indicative of purifying or neutral selection (Doron‐Faigenboim, Stern, Mayrose, Bacharach, & Pupko, 2005). Therefore, the Ka/Ks ratios of orthologous genes between the variety BNZ and the four wild ramie species (BNN, BNW, BAN, and BNT) were used to identify those genes that have been subjected to selection during the domestication of ramie. In this study, we calculated the Ka/Ks ratio of these orthologous genes using the PAML package (Yang, 2007) with default settings.

#### 2.2.4 Testing for introgression

The clean reads of the 15 RNA‐seq datasets mapping to the *Boehmeria nivea* cultivar Zhongzhu No. 1 (Luan et al., 2018) were aligned using HISAT2 software (v2.0.4; http://ccb.jhu.edu/software/hisat2/index.shtml). GATK (McKenna et al., 2010) software was used to detect SNPs, whereas PLINK software (Purcell et al., 2007) was used to convert the output format. Finally, using a cutoff value of minor allele frequency (MAF) higher than 0.05 and an integrity value above 80%, we selected a total of 16,358 SNPs. Based on the pooled SNPs frequency data, both postspeciation gene flow and introgression were analyzed using the program *TreeMix* (version 1.12; Pickrell and Pritchard 2012).

#### 2.2.5 Analysis of differential gene expression in the five ramie transcriptomes

Gene expression levels were initially determined using RSEM (Li & Dewey, 2011). The clean data of 15 separately pooled samples were mapped back onto the assembled ramie transcriptome, and a read count for each gene was obtained from the mapping results. For differential gene expression analysis, the read counts were adjusted using the edge R program package through a one‐scaling normalized factor. Because of the lack of biological replicates for each sequenced library, the read counts were initially adjusted by the edge R program package through a one‐scaling normalized factor. Differential expression analysis of two samples was then performed using the DEGseq R package (Wang, Feng, & Wang, 2010). Thereafter, the *p*‐value was adjusted using *q* value (Storey and Tibshiran 2003). The thresholds *q* value < 0.005 and |log2(foldchange)| >1 were set to define significantly differential expression.

We compared variation in the BNZ transcriptome with that of the four wild ramie species with respect to leaf, stem, and floral tissues to identify differentially expressed genes. These differentially expressed genes were subsequently used for the GO term/KEGG pathway enrichment analyses. GO enrichment implemented using the GOseq R packages based on Wallenius noncentral hyper‐geometric distribution (Young, Wakefield, & Smyth, 2010), and KOBAS software (Mao, Cai, & Olyarchuk, 2005) was used to examine the statistical enrichment of differentially expressed genes in KEGG pathways. We also annotated these genes based on Nr (*E*‐value ≤ 1E‐5), Nt (*E*‐value ≤ 1E‐5), Pfam (*E*‐value ≤ 0.01), KOG/COG (*E*‐value ≤ 1E‐3), Swiss‐Prot (*E*‐value ≤ 1E‐5), KO (*E*‐value ≤ 1E‐10), and GO (*E*‐value ≤ 1E‐6) databases.

## 3 RESULTS

### 3.1 Assembly of the all‐ramie transcriptome

We initially assembled an all‐ramie transcriptome based on the 15 sequencing databases (Table S1) using Trinity (Grabherr et al., 2011) and obtained 182,534 transcripts. By taking the longest transcript of each gene as a representative unigene, we obtained 119,114 unigenes, which is significantly larger than the number of unigenes in the transcriptome of the individual subspecies. Further, the average length of the unigenes (633 bp) and N50 (1,115 bp) in the all‐ramie transcriptome was also slightly increased (Figure S1). Within the all‐ramie transcriptome, the length of unigenes varied from 201 to 16,088 bp, with more than half of the unigenes (69.4%) ranging in size from 200 to 500 bp, and approximately 15.9% being longer than 1,000 bp. These results indicated that this ramie transcriptome could provide a set of long transcripts of ramie. Additionally, we assembled five transcriptomes (BAN, BNT, BNN, BNW, and BNZ) with N50 values of 1,579, 1,524, 1,548, 1,432, and 1,522 bp, respectively.

### 3.2 Functional annotation and classification of the all‐ramie unigenes

To obtain more comprehensive transcript information, we performed functional annotation of the unigenes using BLASTx against different public databases. Approximately 41.31% (49,214), 32.71% (38,969), 40.02% (47,679), 18.76% (22,357), 36.34% (43,287), 36.95% (44,024), and 23.31% (27,773) of the unigenes were perfectly matched to the Nr, Nt, Swiss‐Prot, KEGG, PFAM, GO, and COG databases, respectively (Table S2). A total of 68,145 (57.02%) unigenes were identified in at least one database (Table S2). The remaining 50,969 (42.98%) unigenes matched none of the genes in the public databases searched in this study.

**Figure 1:**
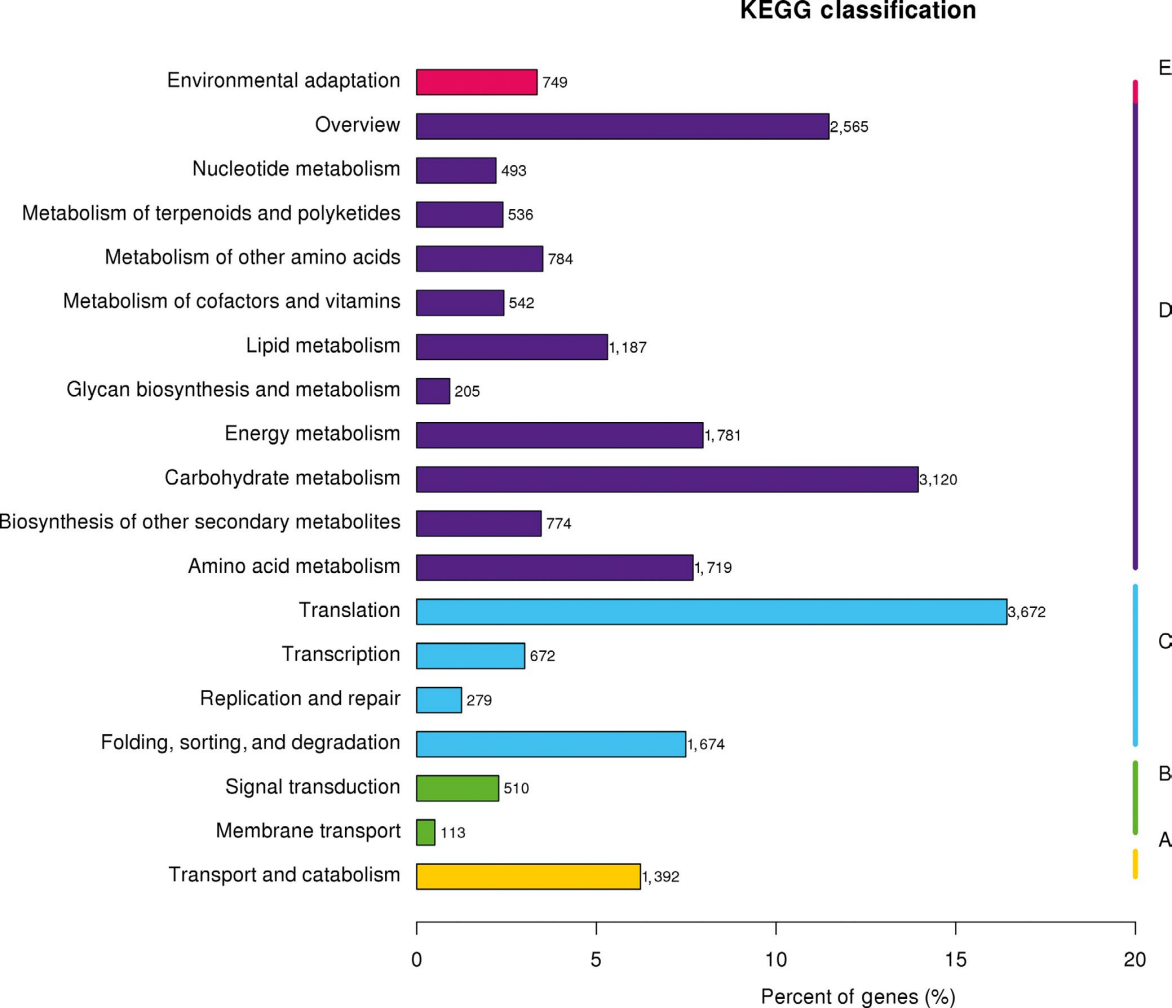
KEGG classification of the all‐ramie transcriptome

GO analysis showed that 44,024 annotated unigenes were assigned to 56 terms of the three main categories: molecular function (22.84%, 50,717), biological process (47.71%, 105,925), and cellular components (29.45%, 65,377) (Figure S2). By searching the COG database, 27,773 (23.31%) unigenes could be clustered into 26 functional categories (Figure S3). Furthermore, through matching to the KEGG database, 22,357 unigenes (18.76%) were assigned to five main categories (cellular processes, environmental information processing, genetic information processing, metabolism, and organismal systems), including 131 KEGG metabolic pathways (Figure 1). The information annotated by the GO, COG, and KEGG databases provided a comprehensive blueprint of the ramie transcriptome and facilitated our investigation of the biological functions, biological processes, and metabolic pathways associated with ramie genes.

### 3.3 Phylogenetic tree construction and testing for introgression

**Figure 2:**
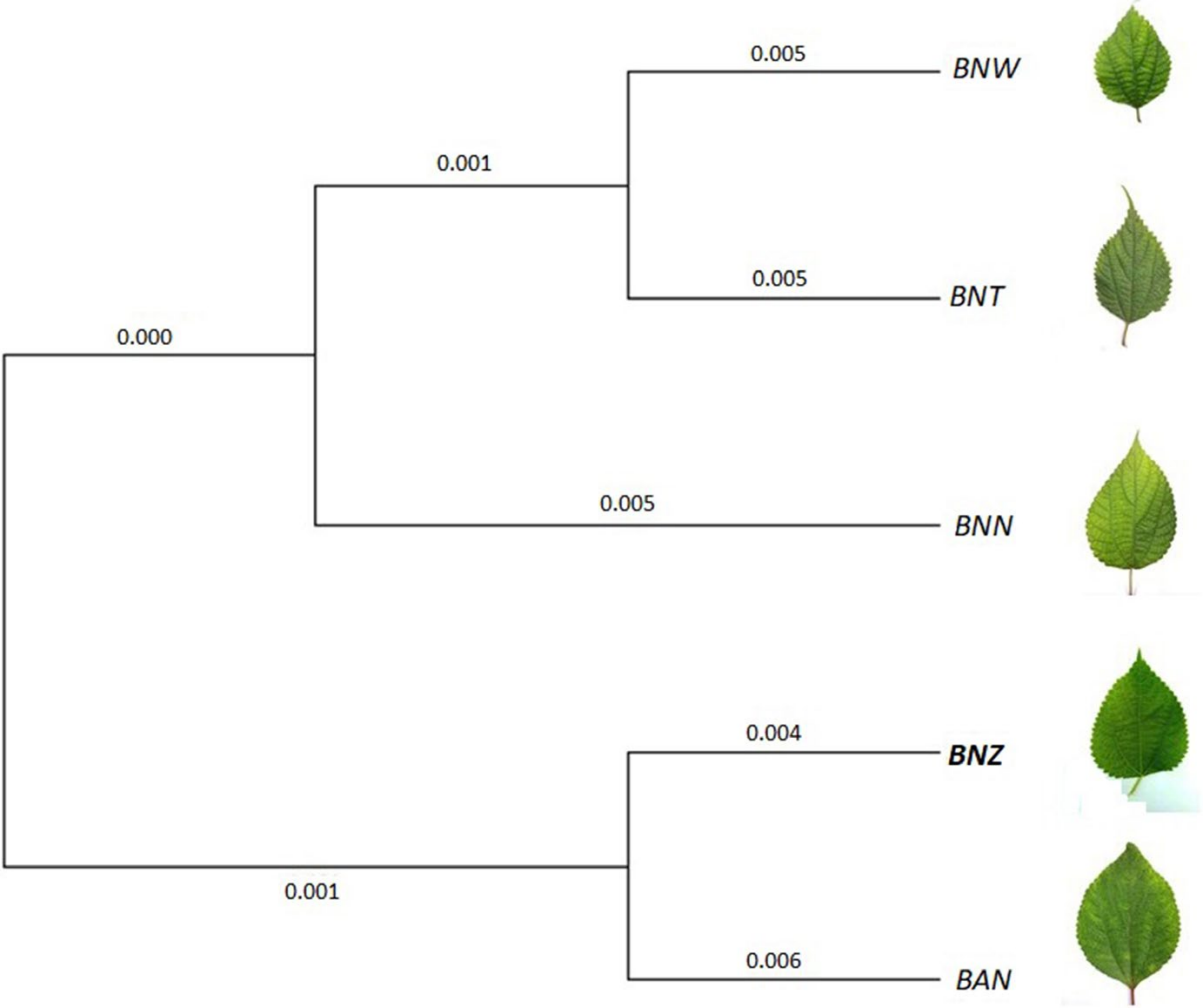
Phylogenetic tree of five ramie species. BNT (*B. nivea* var. *tenacissima*); BNW (*B. nivea* var. *nivea*); BNN (*B. nivea* var. *nipononivea*); BAN (*B. nivea* var. *viridula*); BNZ (cultivar *B. nivea*‐ZZ1)

Using 7,084 orthologous unigene pairs (Table S3), we performed CDS sequence alignment of one‐to‐one orthologous genes using Muscle with default parameters. A phylogenetic tree was constructed based on the maximum likelihood of the PhyML program using the neighbor‐joining (N‐J) technique. The constructed phylogenetic tree showed that the cultivar BNZ was most closely related to BAN, with which it clustered in a group, whereas BNW was firstly clustered with BNT, then BNN formed a separate group (Figure 2).

**Figure 3:**
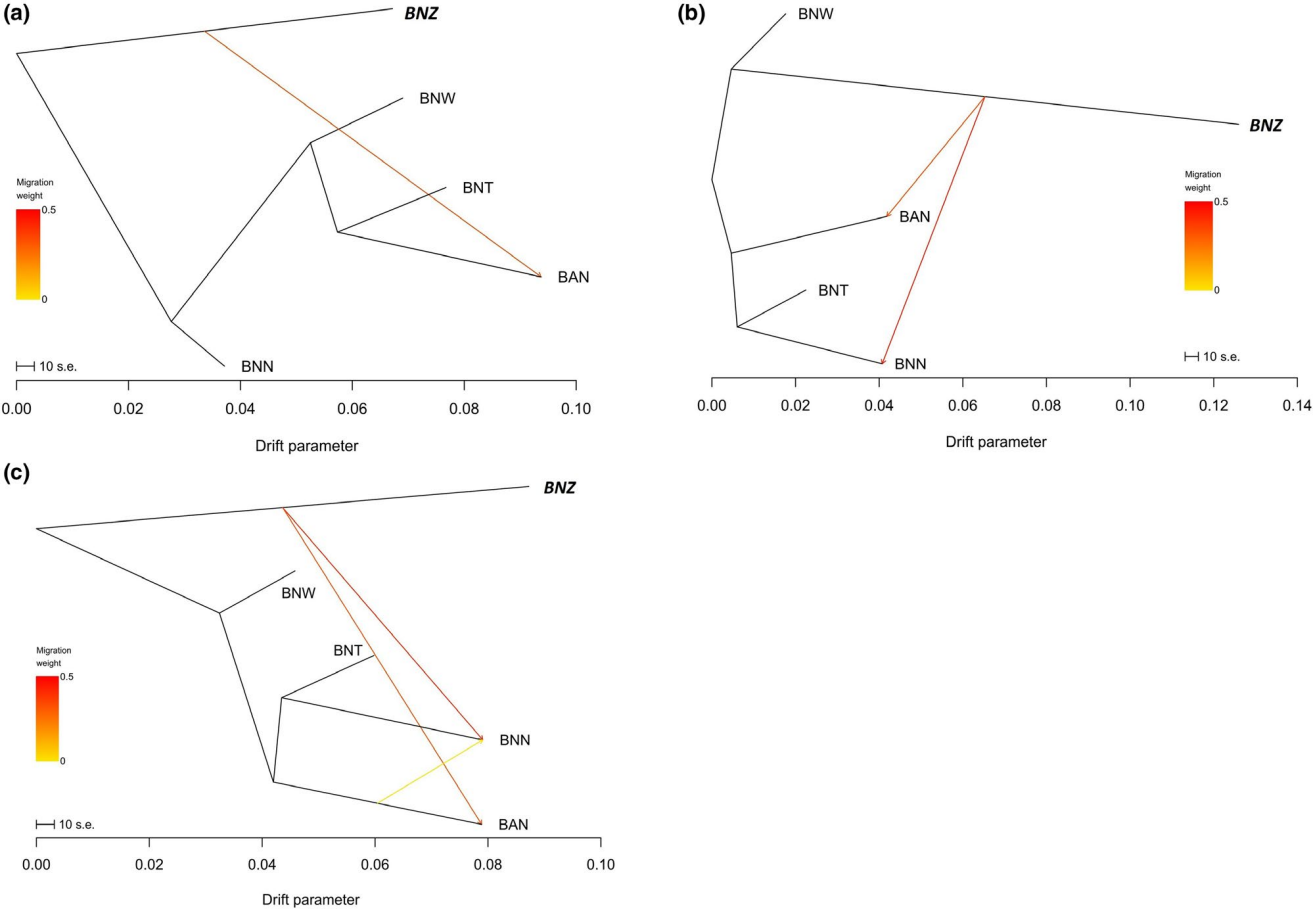
Gene flow of the four wild species and the ramie cultivar ZZ1 using the software *TreeMix*. (a): a one gene flow event analysis; (b): a two‐introgression‐event analysis; (c): a three‐introgression‐event analysis

Based on the pooled 16,358 SNPs, we set up one, two, and three gene flow events using *TreeMix* software (version 1.12; Pickrell & Pritchard, 2012). Our results showed that BNZ and BAN are related when compared in a one gene flow event analysis; a two‐introgression‐event analysis demonstrated the existence of gene flow from BNZ to wild BNN and BAN, and from BAN to BNN according to a three‐introgression‐event analysis (Figure 3). From the three introgression events, it was clear that the gene migration weight of BNZ to BNN was higher than that to BAN, and the gene flow of BAN to BNN (Figure 3c).

### 3.4 KA/KS analysis between domesticated ramie BNZ and the four wild ramie species

The coding gene Ka/Ks ratio can be used to estimate selective pressure. In this study, we found that 2,425 and 269 unigenes were subjected to significant purifying and positive selection, respectively (*Q* value < 0.05) (Tables S4 and S5). On the basis of the corrected p‐value (*p* < 0.05), we found 16 significantly enriched GO terms for these selected genes, including the terms: non‐membrane‐bounded organelle (GO:0043228), intracellular non‐membrane‐bounded organelle (GO:0043232), purine ribonucleoside triphosphate binding (GO:0035639), small molecule binding (GO:0036094), nucleoside binding (GO:0001882), anion binding (GO:0043168), nucleotide binding (GO:0000166), purine ribonucleoside binding (GO:0032550), and structural molecule activity (GO:0005198) (Table S6), indicating that these unigenes play key roles in ramie growth and development. However, no significantly enriched GO terms were found for the unigenes in Table S7. Among these enriched GO terms, 13 genes were linked to the oxidation–reduction process (GO:0055114), 11 to stress response pathways (GO:0006950), 3 to photosystems (GO:0009521), 2 to heat shock protein binding (GO:0031072), 2 to cell development (GO:0048468), 2 to hormone activity (GO:0005179), 2 to response pathways to wounding (GO:0009611), 2 to pathogenesis (GO:0009405), 1 to growth factor activity (GO:0008083), and 1 to the response to auxin stimulus (GO:0009733).

Further annotation of positively selected genes showed that 16 genes displayed a potential function of stress resistance, and among these, six genes were related to disease resistance, such as OG12314 (TMV resistance protein N‐like) and OG13287 (a wall‐associated receptor kinase); two genes were annotated as disease resistance proteins (OG12370, OG12389); three genes (OG12334, OG07829, OG13012) were involved in metal ion binding or transport; and three chaperone protein genes (OG07557, OG02983, OG08532) and five genes (OG02859, OG08828, OG09495, OG07023, OG08705) were annotated as a cold‐regulated gene, heat stress transcription factor, late embryogenesis abundant protein, heat shock protein binding, and U‐box domain‐containing protein (Table S5). Furthermore, the following 10 genes were subjected to positive selection and may be related to vegetative growth‐associated traits: OG12735 encodes for a sugar transporter protein; OG04074 encodes for an expansin protein; OG08139 encodes for bark storage protein A; OG01979 and OG07691 encode for MYB family transcription factors; OG12478 encodes for a UDP‐glucosyltransferase family 1 protein; OG01665 encodes for a photosystem II 10‐kDa polypeptide; OG03476 annotated as a MADS‐box protein FLC subfamily member; OG06265 annotated as FRIGIDA‐like protein 1; and OG05543 encodes for the flowering‐promoting factor 1‐like protein 1 (Table S5).

### 3.5 Divergence in gene expression in wild and cultivated ramie

#### 3.5.1 Identification and functional annotation of differentially expressed genes

Using the criterion of |Log2(fold change)| ≥1, we identified 430, 511, and 97 differentially expressed genes in the stem, leaf, and floral tissues of ramie, respectively (Table 1). From a comparison of transcriptome BNZ with that of the four wild species, we identified 134 genes commonly upregulated (Table S8). Many of these genes have not yet been characterized in ramie, but the annotated genes included the ramie homolog of the *Malus domestica* transcription factor *bHLH92* (c68430_g1), the *Ricinus communis* TMV resistance protein N (c78913_g1), the *Vitis vinifera* growth‐regulating factor 3 (c75070_g5), the *Morus notabilis* auxin response factor 3 partial mRNA (c28067_g1), the disease resistance protein RPM1 (c87326_g3), the GRF zinc finger gene (c64171_g1), beta‐tubulin gene (c76177_g1), gamma‐tubulin complex component 5 (c88197_g2), and alpha‐tubulin (c77460_g1), indicating that abiotic and biotic stresses and cytoskeletal developmental have played a major role in driving transcriptional variation among these species (Table S8).

**Table 1:** Numbers of genes with differential expression between BNZ and the four wild ramie species

Samples	Tissues	Upregulated	Downregulated
BNZ versus BAN	Stem	842	800
	Leaf	1,522	1,729
	Floral	1,440	1,128
BNZ versus BNT	Stem	1,593	1,473
	Leaf	2,166	2,237
	Floral	743	798
BNZ versus BNN	STEM	1,797	1,448
	Leaf	1,637	1,801
	Floral	643	625
BNZ versus BNW	Stem	627	759
	Leaf	1,202	749
	Floral	460	720
Commonly^a^	Stem	274	156
	Leaf	511	153
	Floral	79	18

^a^ The commonly upregulated or downregulated genes comparing BNZ transcriptome with that of the four wild ramie species [(BNZ/BAN)∩(BNZ/BNN)∩(BNZ/BNT)∩(BNZ/BNW)].

#### 3.5.2 GO and KEGG enrichment of the differentially expressed genes of BNZ versus the four wild ramie species

Among the DEGs (differentially expressed genes) of stem and leaf tissues, GO term enrichment analysis identified genes involved in catalytic activity (GO: 0003824) in domesticated BNZ comparisons with the four wild species, and the cellular glucan metabolic process (GO:0006073), glucan metabolic process (GO:0044042), and carbohydrate metabolic process (GO:0005975) in domesticated BNZ in comparison with BNT, BNN, and BNW (Tables S9, S10). In addition, cellulose synthase activity (GO:0016759), cellulose synthase (UDP‐forming) activity (GO:0016760), and the nucleotide sugar metabolic process (GO:0009225) were enriched in the stem DEGs of domesticated BNZ compared with BAN and BNN; the photosynthesis (GO:0015979), photosystem II oxygen evolving complex (GO:0009654), and photosynthetic membrane (GO:0034357) were enriched in leaf DEGs of domesticated BNZ compared with that of BNN. When analyzing floral DEGs, the genes associated with the catalytic activity (GO: 0003824), cellular glucan metabolic process (GO:0006073), glucan metabolic process (GO:0044042), starch metabolic process (GO:0005982), sucrose metabolic process (GO:0005985), and carbohydrate metabolic process (GO:0005975) were enriched in domesticated BNZ compared with that of BAN, BNT, and BNN; the photosynthesis (GO:0015979), photosynthetic membrane (GO:0034357), and photosystem (GO:0009521) were enriched in domesticated BNZ compared with that of BNW (Table S11). As an excellent cultivar variety, BNZ exhibits a longer, wider stem with thicker bark and higher fiber production than that of wild species (Wu et al. 2003; Liu et al., 2014). The enrichment of these categories is in accordance with that expected from selective pressures for fiber production during ramie domesticated.

**Figure 4:**
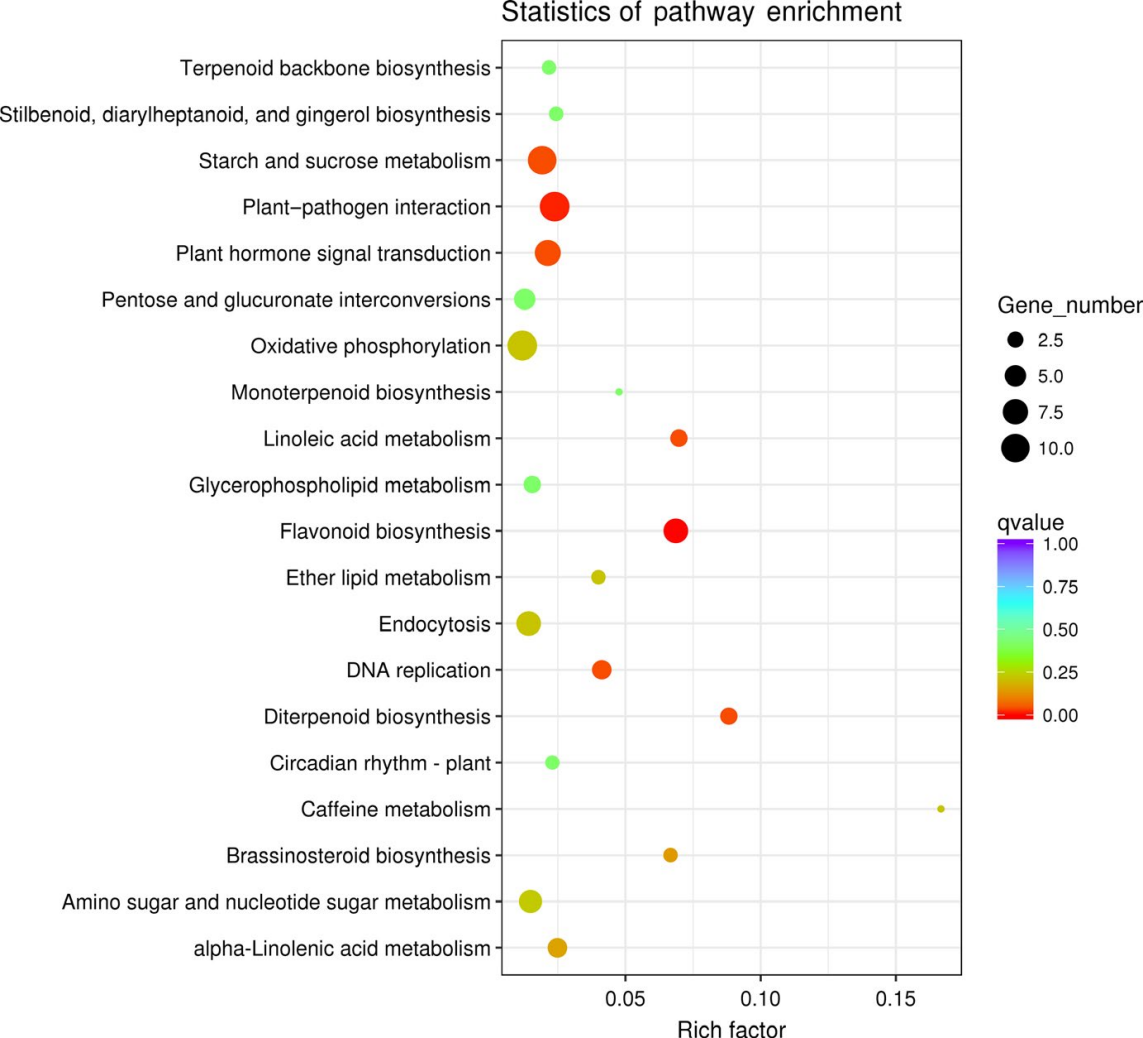
The top 20 KEEG enrichment pathways of stem DEGs comparing cultivar BNZ to four wild ramie species

A KEGG enrichment analysis showed an overrepresentation of genes involved in flavonoid biosynthesis, starch metabolism, sucrose metabolism, cutin, suberine and wax biosynthesis, amino sugar and nucleotide sugar metabolism, and plant hormone signal transduction pathways in the stem, leaf, and floral tissue of BNZ compared with that of the four wild species (Figure 4, Tables 2, S12, S13). In addition, the genes related to the amino sugar and nucleotide sugar metabolic pathway were enriched in BNZ compared with that of BNT and BNN; those related to plant–pathogen interaction were enriched in BNZ compared with that of BAN; and the brassinosteroid biosynthesis pathway was enriched in BNZ compared with that of BNW (Table 2).

**Table 2:** KEGG significantly (*q* value < 0.05) enriched pathways of stem DEG in comparison of domesticated BNZ to that of the four wild species

Samples	KEGG terms	rich_factor	*q* value	gene_number
BNZ/BAN	Plant‐pathogen interaction	0.037422037	0.020311609	18
BNZ/BNT	Starch and sucrose metabolism	0.117082534	6.02E‐09	61
	Plant hormone signal transduction	0.117021277	1.80E‐06	44
	Flavonoid biosynthesis	0.205882353	2.38E‐06	21
	Circadian rhythm—plant	0.195402299	7.00E‐05	17
	Stilbenoid, diarylheptanoid and gingerol biosynthesis	0.195121951	0.000117514	16
	Isoquinoline alkaloid biosynthesis	0.163461538	0.000349231	17
	Cyanoamino acid metabolism	0.155172414	0.000349231	18
	Amino sugar and nucleotide sugar metabolism	0.089974293	0.002424642	35
	Phenylpropanoid biosynthesis	0.082429501	0.005770352	38
	Tyrosine metabolism	0.106145251	0.013804464	19
	DNA replication	0.12371134	0.040086466	12
	alpha‐Linolenic acid metabolism	0.103225806	0.040086466	16
BNZ/BNN	Starch and sucrose metabolism	0.13243762	9.41E‐12	69
	Flavonoid biosynthesis	0.245098039	1.67E‐08	25
	Amino sugar and nucleotide sugar metabolism	0.118251928	1.35E‐06	46
	Isoquinoline alkaloid biosynthesis	0.201923077	4.61E‐06	21
	Photosynthesis	0.183333333	8.01E‐06	22
	Plant hormone signal transduction	0.106382979	6.68E‐05	40
	Stilbenoid, diarylheptanoid, and gingerol biosynthesis	0.195121951	0.000132545	16
	Photosynthesis—antenna proteins	0.266666667	0.000132545	12
	Phenylpropanoid biosynthesis	0.093275488	0.000388466	43
	Tyrosine metabolism	0.12849162	0.000473852	23
	Phenylalanine metabolism	0.108695652	0.000492712	30
	Circadian rhythm—plant	0.172413793	0.000591247	15
	Diterpenoid biosynthesis	0.235294118	0.006658587	8
	Gap junction	0.118421053	0.006658587	18
	Porphyrin and chlorophyll metabolism	0.15942029	0.010024084	11
	Other glycan degradation	0.19047619	0.019448895	8
	Cyanoamino acid metabolism	0.120689655	0.020476113	14
	Osteoclast differentiation	0.129411765	0.038132449	11
BNZ/BNW	Flavonoid biosynthesis	0.156862745	1.23E‐08	16
	Starch and sucrose metabolism	0.047984645	0.000244277	25
	Linoleic acid metabolism	0.162790698	0.000931327	7
	Plant hormone signal transduction	0.050531915	0.001015823	19
	alpha‐Linolenic acid metabolism	0.064516129	0.01305533	10
	Diterpenoid biosynthesis	0.147058824	0.01305533	5
	Metabolism of xenobiotics by cytochrome P450	0.065693431	0.015609717	9
	Sesquiterpenoid and triterpenoid biosynthesis	0.1	0.015609717	6
	Drug metabolism—cytochrome P450	0.063829787	0.015609717	9
	Chemical carcinogenesis	0.060150376	0.036439475	8
	Amino sugar and nucleotide sugar metabolism	0.038560411	0.036439475	15
	Brassinosteroid biosynthesis	0.133333333	0.036439475	4

## 4 DISCUSSION

### 4.1 The phylogenetic relationships among members of the section Tilocnide and introgression

The phylogenetic analysis showed that BAN clusters with the cultivar BNZ in one group, BNW is closely related to BNT, and BNN forms a separate group. However, this differed from previous reports wherein BAN was considered to be the most primitive taxon, and in which BNT evolved into either *B. nivea* var. *nivea* (BNW and cultivars) or BNN (Jiang & Jie, 2005; Zhang et al., 1998; Chen, Pei et al., 2014). The findings of Jiang et al. (2005) and Zhang et al. (1998) were based on the evolutionary trends of stipules, leaf hairs, and pollen external morphology. The results of Chen, Pei et al. (2014) were based on cluster analysis of 18 morphological characteristics of ramie. These results may fail to represent the phylogenetic relationships among members of the section Tilocnide because of environmental impacts on the phenotype. However, to date, the phylogenetic relationships among members of section Tilocnide based on molecular‐level analyses have rarely been reported. Our results differ from those reported by Chen, Pei et al. (2014) and Zhang et al. (1998) but are based on a larger dataset.

We identified gene flow from BNZ to BNN and BAN and between BAN and BNN. It is suggested that BNZ is closely related to BNN and BAN, and that BNN is closely related to BAN. According to our results, based on SNPs annotation from RNA‐seq data, further whole‐genome sequence SNPs may be needed to validate our hypothesis.

### 4.2 Abiotic and biotic stresses and fiber development were important selective pressures in ramie domestication

Among these 269 positively selected genes, 13 genes were involved in the oxidation–reduction process (GO:0055114), 11 genes in stress response pathways (GO:0006950), 2 genes in heat shock protein binding abilities (GO:0031072), and 2 in response to wounding mechanisms (GO:0009611). Modulation of cellular redox status was important during both cotton fiber evolution and improvement (Chaudhary et al., 2008; Hovav et al., 2008; Guo, Du et al., 2016; Guo, Liu et al., 2016), and domestication of cultivars appears to enhance modulation of cellular redox levels, avoid or delay stress, and prolong the duration of fiber elongation (Yoo & Wendel 2014). Similarly, as a bast fiber crop, the duration of fiber elongation was important for ramie fiber improvement, and a previous study (Meng, Wu, Zhou, & Sun, 2013) showed that fiber cell length was significantly longer in domesticated BNZ than in wild species. Thus, it is likely that the modulation of cellular redox status was important during ramie domestication. Several genes deserve to be highlighted. The cell wall‐associated receptor kinase gene (OG13287), as a candidate receptor from oligogalacturonides, was active in damage‐associated molecular patterns and elicited a plant immune response (Brutus, Sicilia, Macone, Cervone, & Lorenzo, 2010); it plays a crucial role in protecting plants against stress by re‐establishing normal protein conformation and cellular homeostasis (Wang, Vinocur, Shoseyov, & Altman, 2004). In addition, three chaperone protein genes (OG07557, OG02983, OG08532), one cold‐regulated gene (OG02859), one heat stress transcription factor (OG08828), one late embryogenesis abundant protein (OG09495), and a heat shock protein binding (OG07023) play a role in the protection of tissues against oxidative damage (Park et al. 2015; Eremina, Rozhon, & Poppenberger, 2016; Guo, Du et al., 2016; Guo, Liu et al., 2016; Wang et al., 2004). A TMV resistance protein N‐like (OG12314) and two disease resistance protein genes (OG12370 and OG12389) were under positive selection, which have been reported to take part in the defense response to pathogens (Hehl et al., 1999; Martin et al. 2003).

Furthermore, several genes involved in resistance to abiotic or biotic stresses were upregulated in domesticated BNZ compared with that of the four wild species, such as a transcription factor *bHLH92*, a TMV resistance protein N, a disease resistance protein RPM1, and a GRF zinc finger gene. In *Arabidopsis thaliana*, the overexpression of *bHLH92* moderately increased the tolerance to NaCl and osmotic stresses (Jiang, Yang, & Deyholos, 2009). The TMV resistance N‐like protein is involved in *Synchytrium endobioticum* resistance in potatoes (Hehl et al., 1999). The disease resistance protein RPM1 confers resistance to *Pseudomonas syringae* in *Arabidopsis thaliana* (Boyes, Nam, & Dangl, 1998). Coupled with the preceding results of positive selection genes, these results strengthen our working hypothesis regarding the abiotic and biotic stresses during artificial selective pressures in ramie domestication, which are consistent with the finding of Liu et al. (2014).

In addition, cortical microtubules provide spatial information necessary for the alignment of cellulose microfibrils that confine and regulate cell elongation (Whittaker et al. 1999). Alpha‐tubulin was found with specific transcript accumulation in developing cotton fibers (Whittaker et al. 1999), and beta‐tubulin was upregulated in domesticated cottons (Yoo & Wendel 2014; Rapp et al., 2010). Similarly, we found a beta‐tubulin gene (c76177_g1), gamma‐tubulin complex component 5 (c88197_g2), and an alpha‐tubulin (c77460_g1) that were more highly expressed in domesticated ramie, and it is likely ramie fiber differentiation requires dynamic cytoskeletal activity as does cotton.

### 4.3 Vegetative growth‐associated genes under positive selection during ramie domestication

Unlike cotton fibers, which are an epidermal seed fiber, ramie fiber originates from the phloem tissue of stems, a vegetative organ. Therefore, vegetative growth‐associated traits may have been prominent factors selected during the artificial domestication process (Liu et al., 2017). In the present study, one gene (OG03476) homolog of the *Coffea arabica* MADS‐box protein FLC subfamily gene, a gene (OG06265) homolog of *Vitis vinifera* FRIGIDA‐like protein 1, and a gene (OG05543) annotated as a flowering‐promoting factor 1‐like protein 1 were under positive selection during ramie domestication. Both FLC and its upstream regulator FRIGIDA (FRI) are major determinants of natural variation in flowering time (Michaels et al. 1999; Adrian, Torti, & Turck, 2009; Choi et al., 2011). *FLC* interacts with another MIKC^C^‐type floral repressor, SHORT VEGETATIVE PHASE (SVP), repressing the expression of the mobile floral inducer *FLOWERING LOCUS T* (FT) and other genes that initiate floral transition (Li et al., 2008; Adrian et al., 2009). Furthermore, *FLC* is involved in developmental processes, including the juvenile‐to‐adult transition and floral organ development (Deng et al., 2011). Domestication‐related flowering time variation by means of mutations in orthologs of FLOWERING LOCUS C (FLC) or its upstream regulator FRIGIDA (FRI) is typical of members of the Brassicaceae lineage (Wu et al., 2012; Okazaki et al., 2007). Early flowering automatically reduces the time for vegetative growth, thus leading to smaller plants, whereas late flowering increases it, resulting in larger plants (Lenser et al. 2013). Given the strong selection for the vegetative growth‐associated traits in ramie, such as plant height, stem diameter, and bast thickness–as showcased by the morphological differences in the cultivated variety–these three genes are good candidates for the alteration of the flowering time and the promotion of a longer vegetative growth period.

## CONFLICT OF INTEREST

The authors declare that they have no competing interests.

## AUTHOR CONTRIBUTIONS

KH, ML, and JC conceived and designed the experiments; KH, AZ, XC, YS, QT, XW, and ZS performed the experiments; and KH, ML, and JC analyzed the data and wrote the paper.

## Data Availability

The raw sequencing reads have been deposited in the NCBI Sequence Read Archive (SRA, https://www.ncbi.nlm.nih.gov/Traces/sra) with accession numbers SRP150685: SRX4224255‐SRX4224269. SRA records are accessible with the following link: https://www.ncbi.nlm.nih.gov/sra/ SRP150685.
