# Supplementary figures and images for: Comparative transcriptomics reveals the selection patterns of domesticated ramie
Source: Ecol Evol. 2019 May 22;9(12):7057–68. doi: 10.1002/ece3.5271 (PMC6662332; doi:10.1002/ece3.5271)

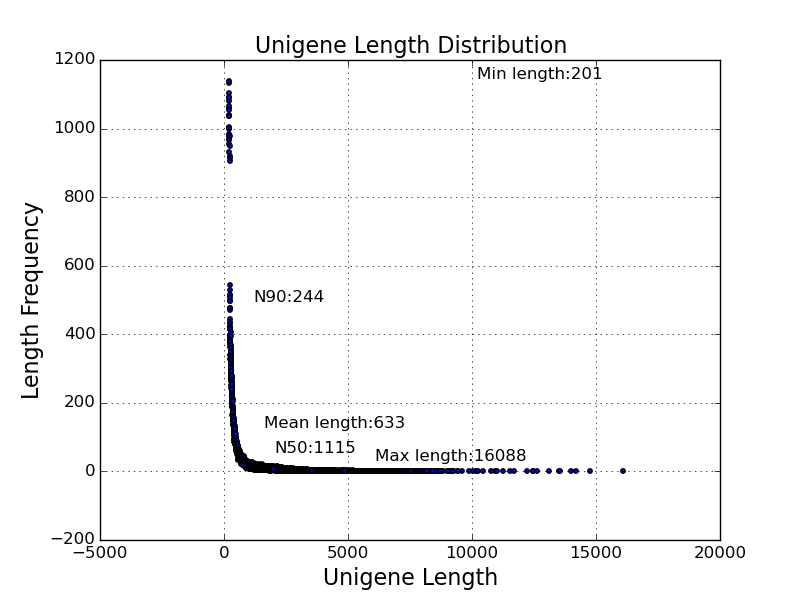

Supplement: Supplementary file 1 [file ECE3-9-7057-s001.png]

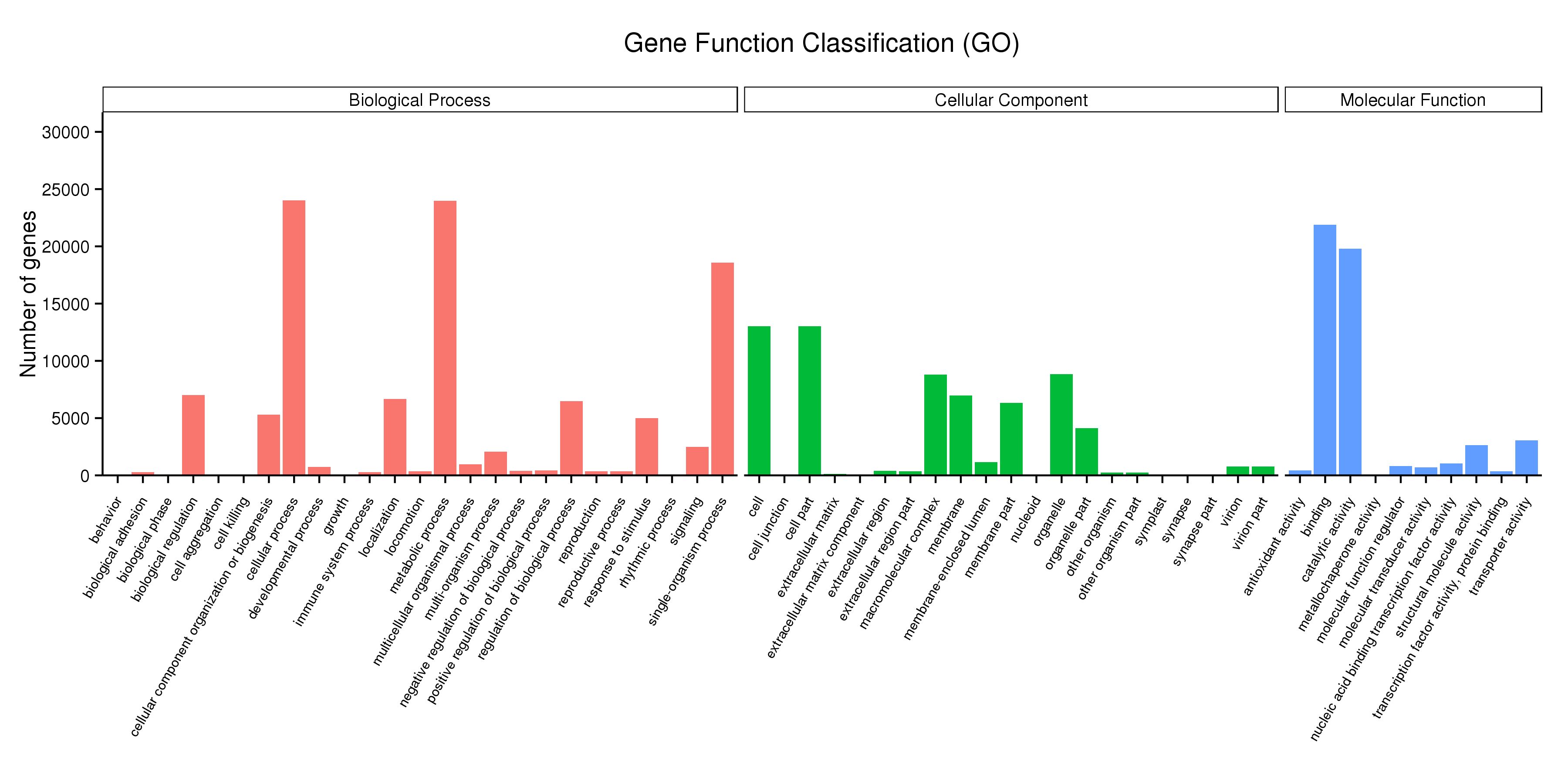

Supplement: Supplementary file 2 [file ECE3-9-7057-s002.png]

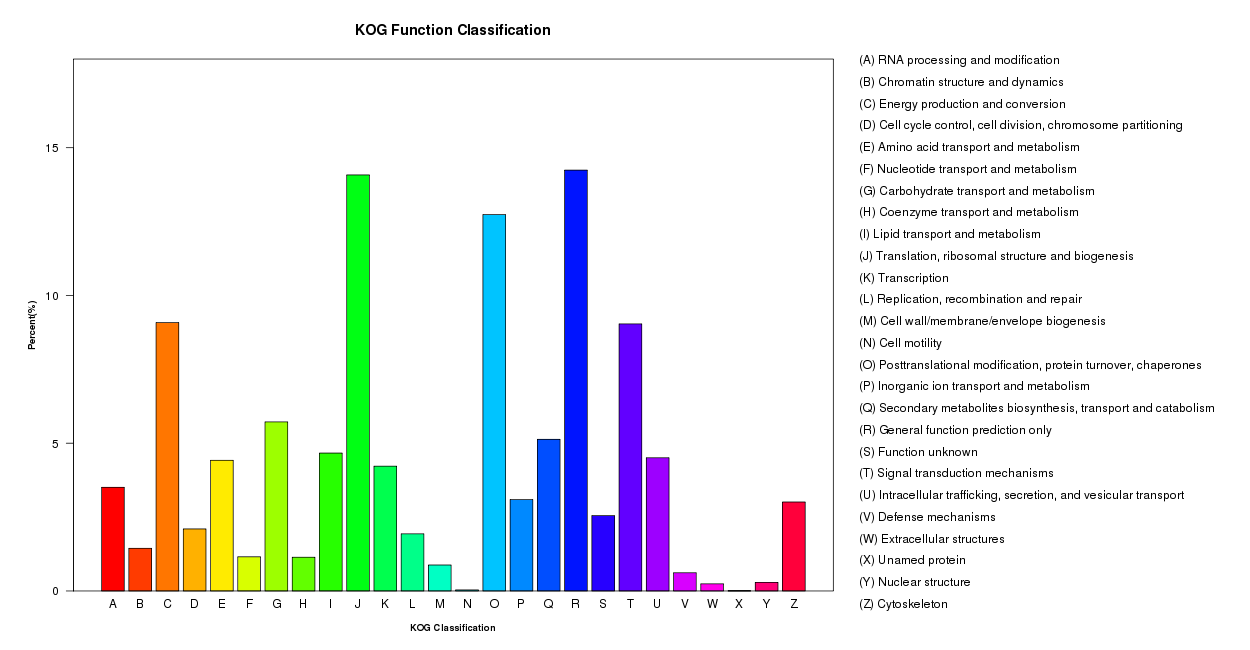

Supplement: Supplementary file 3 [file ECE3-9-7057-s003.png]

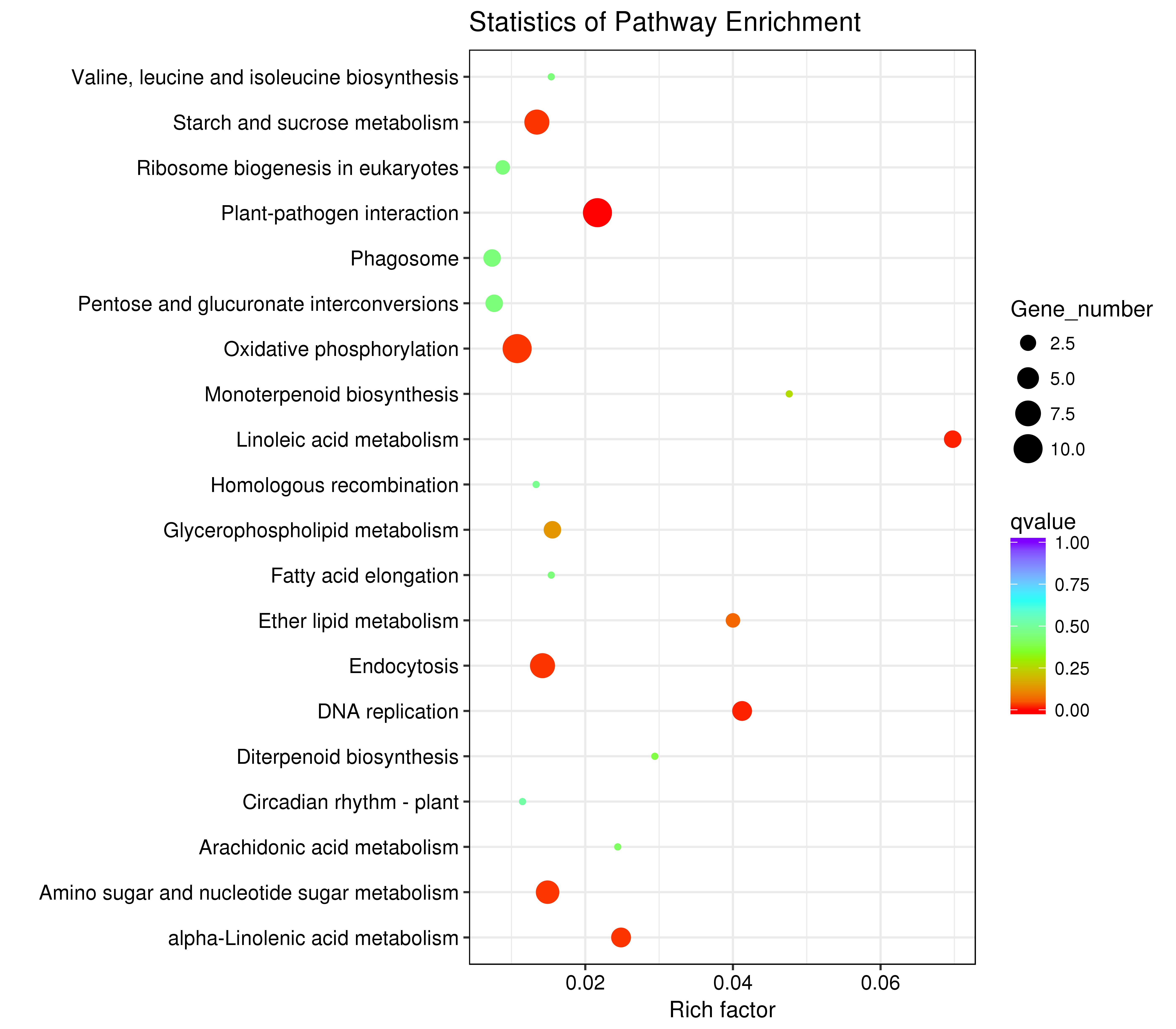

Supplement: Supplementary file 4 [file ECE3-9-7057-s004.png]
